# Nitrogen acquisition in *Agave tequilana* from degradation of endophytic bacteria

**DOI:** 10.1038/srep06938

**Published:** 2014-11-06

**Authors:** Miguel J. Beltran-Garcia, James F. White, Jr., Fernanda M. Prado, Katia R. Prieto, Lydia F. Yamaguchi, Monica S. Torres, Massuo J. Kato, Marisa H. G. Medeiros, Paolo Di Mascio

**Affiliations:** 1 Departamento de Química ICET, Universidad Autonoma de Guadalajara, Patria 1201, Lomas del Valle, Zapopan Jalisco, Mexico; 2 Department of Plant Biology and Pathology, Rutgers University, New Brunswick, NJ USA; 3 Departamento de Bioquímica, Instituto de Química, Universidade de São Paulo, 05508-000, São Paulo, SP, Brazil; 4 Departamento de Química Fundamental, Instituto de Química, Universidade de São Paulo, 05508-000, São Paulo, SP, Brazil

## Abstract

Plants form symbiotic associations with endophytic bacteria within tissues of leaves, stems, and roots. It is unclear whether or how plants obtain nitrogen from these endophytic bacteria. Here we present evidence showing nitrogen flow from endophytic bacteria to plants in a process that appears to involve oxidative degradation of bacteria. In our experiments we employed *Agave tequilana* and its seed-transmitted endophyte *Bacillus tequilensis* to elucidate organic nitrogen transfer from ^15^N-labeled bacteria to plants. *Bacillus tequilensis* cells grown in a minimal medium with ^15^NH_4_Cl as the nitrogen source were watered onto plants growing in sand. We traced incorporation of ^15^N into tryptophan, deoxynucleosides and pheophytin derived from chlorophyll *a*. Probes for hydrogen peroxide show its presence during degradation of bacteria in plant tissues, supporting involvement of reactive oxygen in the degradation process. In another experiment to assess nitrogen absorbed as a result of endophytic colonization of plants we demonstrated that endophytic bacteria potentially transfer more nitrogen to plants and stimulate greater biomass in plants than heat-killed bacteria that do not colonize plants but instead degrade in the soil. Findings presented here support the hypothesis that some plants under nutrient limitation may degrade and obtain nitrogen from endophytic microbes.

Like all living things, plants require nitrogen (N) throughout their development. The N incorporated as NO_3_^−^ and NH_4_^+^ represents about 2% of total plant dry matter, and is a component of proteins, nucleic acids, cofactors, signalling molecules, storage and numerous plant secondary products. The availability of N to plant roots is often an important limiting factor for plant growth. Plants obtain N from nitrogen fixing bacteria and decomposition of dead tissues of both plants and animals by microorganisms^1^,^2^,^3^,^4^,^5^. Moreover, a tiny fraction (0.00024%) of planetary N is available to plants in soils, where plants compete with microbes to absorb N^6^. The atmospheric formation of nitrate by photo-oxidation through the effect of lightning also is minimal^9^,^10^. The limited bioavailability of N, and the need to enhance crop growth, have provoked a continual expansion in use of N fertilizer with from 12 to 104 additional teragrams used each year^7^,^8^. On average only 30–50% of the applied N is taken up by plants, with the remainder being lost to surface run-off, leaching of nitrates and ammonia volatilization^11^. The excessive use of fertilizers is a highly contaminating process^9^ and the energy required to produce N through the Haber-Bosch process requires at least 1% of the world annual energy supply^12^.

Historically, it was thought that plants derived all N nutrition from the inorganic forms of N, NO_3_^−^ and NH_4_^+^. However, it is now known that the principal form of N entering soils that do not receive inorganic fertilizer is organic N derived from microbial breakdown of organic matter, including amino acids, di- and tri-peptides, DNA and proteins^13^,^14^,^15^. In addition, some plants can scavenge organic N from insects (carnivorous plants) or by entomopathogenic fungus-mediated N translocation from insects^16^,^17^. Further, some plants have been shown to consume and degrade bacteria and yeasts through endocytosis^3^. However, it is unclear whether endocytosis of microbes by plants is a purely defensive process or rather is a means to acquire nutrients^3^,^4^,^5^. Plants are naturally colonised by endophytic bacteria that inhabit internal spaces without deleterious effects on host plants^1^,^2^. Endophytic bacteria that spend some time in soil enter roots or shoots and may spread to all plant tissues, often transmitting in seeds, thus ensuring transfer to the next plant generation^2^. However, controversy exists regarding the significance of nitrogen-fixing endophytes in plants^1^,^2^. It is frequently argued that positive effects of bacteria on plant growth may be the result of auxins and other growth regulators rather than enhanced N acquisition due to microbial N fixation^18^,^19^,^20^,^21^. Also there is no clear evidence as to how N is transferred from endophytic diazotrophs to the host plant. It has been suggested that N transfer to the host is via amino acids, ammonia, small organic molecules or mineralization of dead bacterial cells^22^. Whether N is actually transferred from endophytic bacteria to plant host has proven difficult to resolve. The question of the mechanism of transfer of N from endophyte to host plant is an important gap in our knowledge. One possible mechanism for N transfer to host is that plants may scavenge organic nitrogen by oxidation and degradation of endophytic bacteria or their proteins using reactive oxygen species (ROS) to lyse cells and denature proteins. This mechanism has been termed ‘oxidative nitrogen scavenging'^4^,^23^. The experiments reported here were done to evaluate whether ^15^N incorporated into bacterial endophyte biomolecules such as proteins and nucleic acids could be traced into plant molecules; and whether the transfer process involves evidence of oxidative degradation of microbes.

## Results

### ^15^N_2 _gas assimilation into seedlings

Shoots of seedlings grown in ^15^N_2_-enriched air showed higher δ ^15^N vs air content (54.93 ± 5.65 δ ^15^N *vs* (‰); mean ± standard error of mean) than shoots from seedlings grown in non-enriched air (3.07 ± 1.12 δ ^15^N *vs* (‰)). This large difference in the ^15^N/^14^N ratios between the two treatments shows that N was fixed in the seedling tissues.

### *Bacillus tequilensis* endophyte

**Figure 1:**
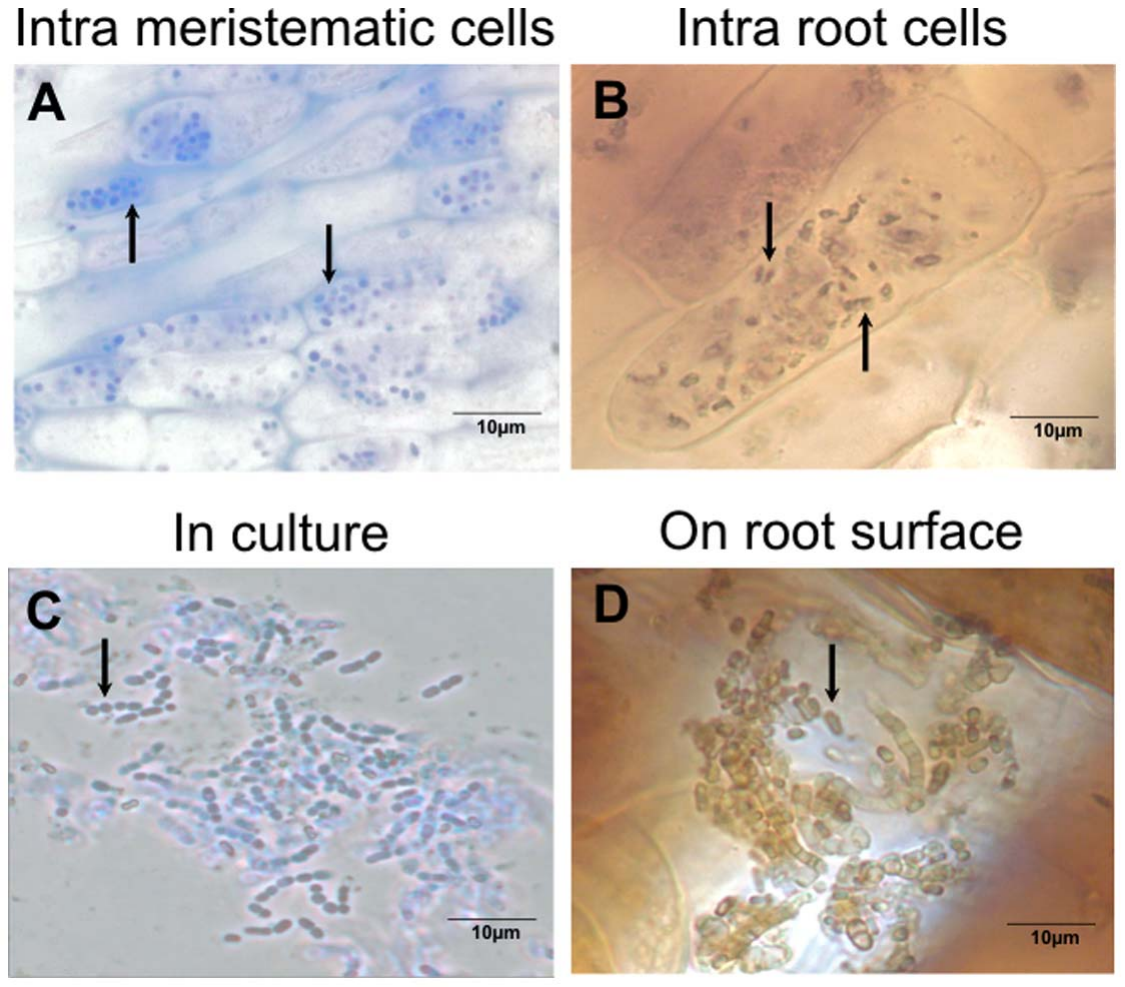
*Bacillus tequilensis*: an endophyte of *Agave tequilana*. Intracellular bacteria in meristematic root cells (A) and oxidation within root cortical cell (B), detail of cell morphology of the isolate grown on TSA medium (C), and bacteria on root surface showing H_2_O_2_ concentrations (brown) around bacterial cells (D).

For use in our tests to evaluate ^15^N transfer/translocation from bacterial endophyte to plant, we isolated an endophyte from seeds, seedlings and young plantlets (bulbillos) of *Agave tequilana* Weber, and identified it as endospore-forming *Bacillus tequilensis* (accession number KF792125)^24^ (Fig. 1) using sequence data. *Bacillus tequilensis* was present in both roots (Fig. 1C) and shoots of the host plant.

### Visualization of bacteria in plant tissues

In seedling infection experiments using *B. tequilensis* we observed intracellular colonization by the bacterium into meristems and root epidermal cells of seedlings (Fig. 1A and 1B) and young plantlets. We further observed oxidation of bacterial cells within and on the surface of root epidermal cells (Fig. 1B and 1D)^25^.

## Experiment 1

### ^15^N tracking experiments

**Figure 2:**
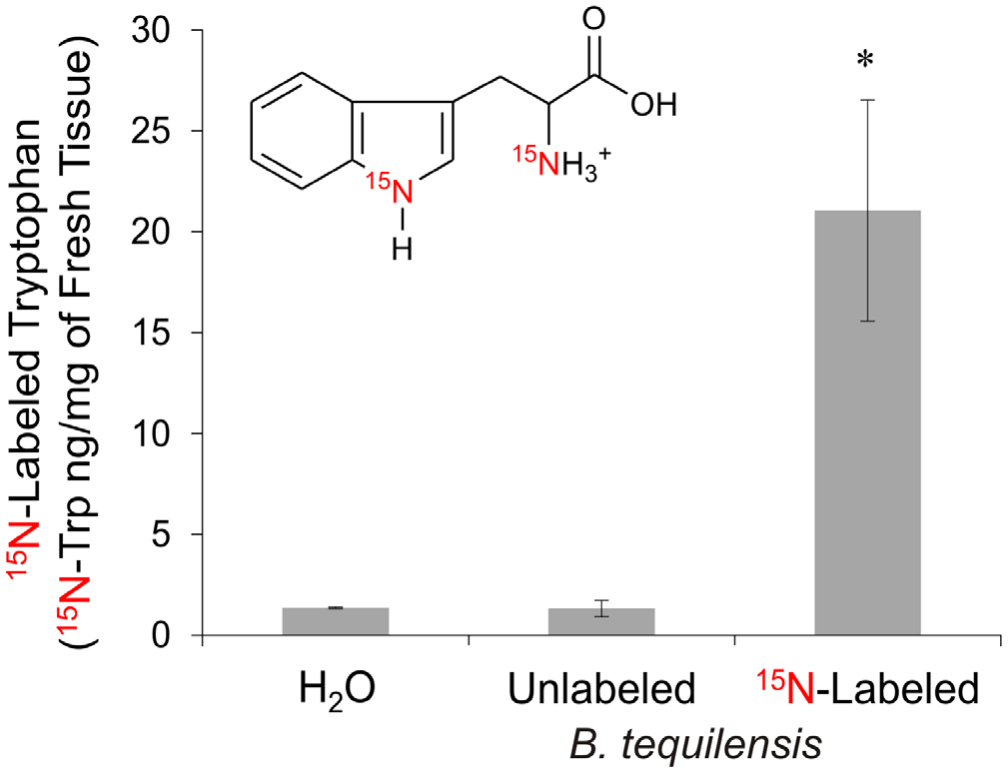
Quantification of ^15^N-Trp in foliar tissue of *A. tequilana* using HPLC-MS/MS. The sample groups analyzed were H_2_O treated, unlabeled *B. tequilensis* (^14^N*-Bteq*) and ^15^N-labeled *B. tequilensis* (^15^N*-Bteq*). Nitrogen content was calculated as the quantity of ^15^N-Trp (ng/mg). Data are the mean values ± standard error of the mean from three independent experiments. (*)^15^N-labeled *B. tequilensis* data are significantly different when compared with the H_2_O and unlabeled *B. tequilensis* groups (p<0.05). For multiple comparisons, t-test was applied.

A significant difference (p<0.05) was observed in ^15^N-labeled tryptophan (^15^N-Trp) concentration between the controls (H_2_O and unlabeled *B. tequilensis*, ^14^N*-Bteq*) and plants inoculated with ^15^N-labeled *B. tequilensis* (^15^N*-Bteq*). ^15^N-labeled Trp in the ^15^N*-Bteq* treated plants was about 16-fold higher (21.05 ± 5.47 ng/mg) than ^14^N*-Bteq* treated (1.32 ± 0.40 ng/mg) and H_2_O treated plants (1.36 ng/mg) (Fig. 2, and Supplementary Fig. S5). Further quantification of ^15^N-Trp demonstrated that plants supplemented with ^15^NH_4_Cl had higher levels than plants supplemented with H_2_O or ^14^NH_4_Cl (Supplementary Fig. S1). We detected tryptophan by high-performance liquid chromatography coupled to mass spectrometry in tandem (HPLC-MS/MS), although this amino acid was not quantified (Supplementary Fig. S4)^26^,^27^,^28^.

**Figure 3:**
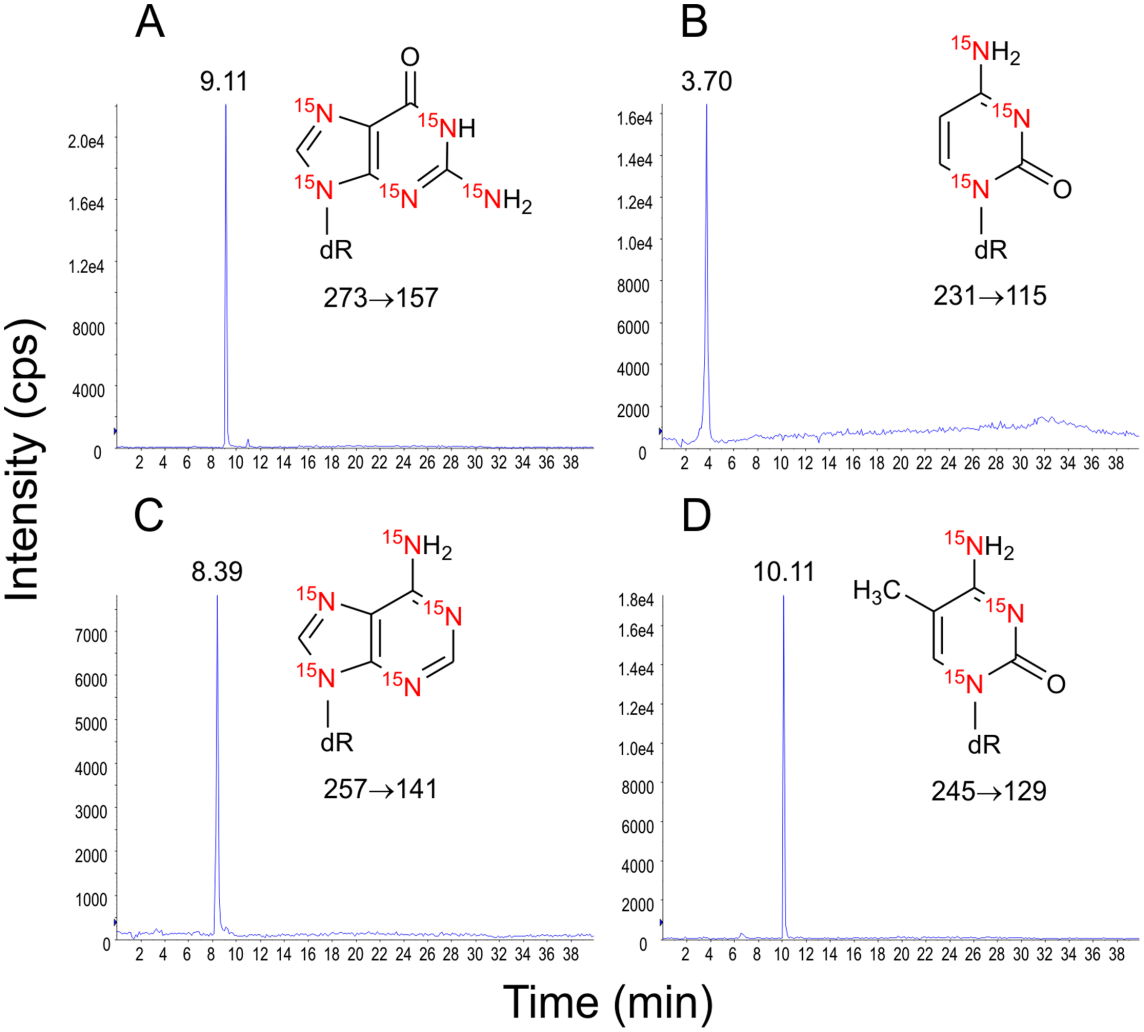
HPLC-MS/MS analysis of ^15^N-labeled 2′-deoxynucleosides from *A. tequilana* supplemented with ^15^N-labeled *B. tequilensis*. 2′-Deoxynucleosides were detected by the loss of the 2-deoxyribose moiety: ^15^N_5_-dG, *m/z* 273→157 (A), ^15^N_3_-dC, *m/z* 231→115 (B), ^15^N_5_-dA, *m/z* 257→141 (C) and ^15^N_2_-dT, *m/z* 245→129 (D).

Nitrogen incorporation into tissues of *A. tequilana* was also confirmed by HPLC-MS/MS detection of ^15^N-labeled nucleosides (Fig. 3). Additionally, the same ^15^N-labeled nucleosides were detected from *A. tequilana* supplemented with ^15^N-labeled NH_4_Cl (Supplementary Fig. S9). Detection of 2′-deoxynucleosides methylated as 5-methyl-2′-deoxycytidine (^15^N_3_- MedC) and *N*′-methyl-2′-deoxyadenosine (^15^N_5_-MedA) was also observed in plants of *A. tequilana* supplemented with ^15^N-labeled *B. tequilensis* or 15N-labeled NH_4_Cl (Supplementary Fig. S10 and 11).

### Pheophytin analysis

**Figure 4:**
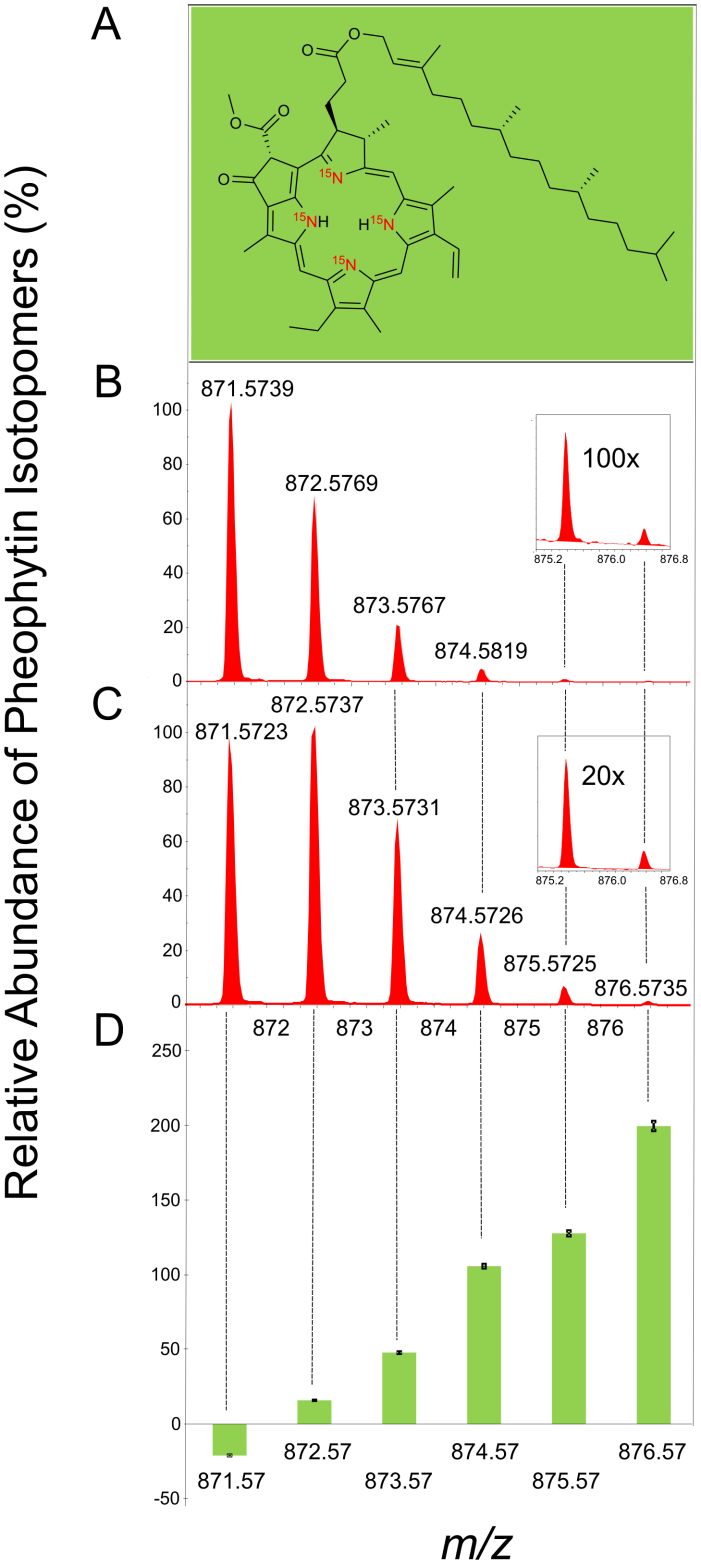
Relative abundance of pheophytin isotopomers from *A. tequilensis*. (A) Chemical structure; (B) Percentage increase of pheophytin isotopomers calculated from plants supplemented with unlabeled *B. tequilensis* (C) and with ^15^N-labeled *B. tequilensis* (D). The data points shown are the mean values ± standard error of the mean from three independent experiments. p<0.08; p<0.02; p<0.05; p<0.01 and p<0.003, respectively for *m/z* 871.57, 872.57, 873.57, 874.57 and 875.57, comparing ^14^N and ^15^N-labeled *B. tequilensis* groups.

Analysis of unlabeled pheophytin *a* (Fig. 4B)^26^,^27^ showed a distribution of isotope peaks in agreement with the theoretical values of this molecular formula (C_55_H_74_N_4_O_5_, [M+H]^+^ = 871.5731) containing a base peak with *m/z* 871.5739 (Fig. 4B). Incubation of *A. tequilana* with the ^15^N-labeled *B. tequilensis* resulted in incorporation of ^15^N into the isotopomers of pheophytin. The relative abundance of the isotopomers *m/z* 872.57, 873.57, 874.57, 875.57, 876.57 increase by 16, 48, 106, 128 and 200% respectively (p<0.08; p<0.02; p<0.05; p<0.01 and p<0.003, respectively for *m/z* 871.57, 872.57, 873.57, 874.57 and 875.57, comparing ^14^N and ^15^N-labeled *B. tequilensis* groups, Fig. 4D and inset 4B and 4C). The ^15^N uptake to form the isotopomer *m/z* 876.57 pheophytin was notably greater, than that of unlabeled leaves, attesting to the incorporation of ^15^N atoms into the tetrapyrrole ring of the pheophytin molecule.

## Experiment 2

### Biomass increases in plants

In this experiment we treated plants with live or heat-killed ^15^N-labeled *Bacillus* (121°C, 10 min); and with MMN solution (see methods), non-endophytic *E. coli* (not labeled) and water as controls. Biomass measurements after two months of treatment showed that the greatest biomass increase was seen in plants treated with living *B. tequilensis*, where mean biomass increase was 1.63 ± 0.28 g (mean ± standard deviation). Plants treated with heat-killed *B. tequilensis* or mineral nutrient solution (MMN) showed biomass increases of 0.83 ± 0.16 g for heat-killed *B. tequilensis*; and 0.52 ± 0.34 g for MMN. Plants treated with non-endophytic *Escherichia coli* showed a biomass increase of 0.71 ± 0.14 g. Plants treated only with water showed the least biomass increase (0.15 ± 0.05 g).

### Comparison of ^15^N absorption from living verses dead *B. tequilensis*

To evaluate the extent to which ^15^N was moving into plant tissues after the death of bacteria in soil or through endophytic colonization, we included in the experiment living and heat-killed *B. tequilensis* that had been labeled with ^15^N. On analysis we found that incorporation of ^15^N into nucleosides, 2′-deoxycytidine (^15^N_3_-dC) and 2′-deoxyadenosine (^15^N_5_-dA) was significantly greater (p<0.05) in plants treated with live *Bacillus* (a.u.: ^15^N_3_-dC 1.13 × 10^2^ ± 13.93; ^15^N_5_-dA 4.50 × 10^1^ ± 9.05) than plants treated with the heat-killed *Bacillus* (a.u.: ^15^N_3_-dC 3.40 × 10^1^ ± 26.51; ^15^N_5_-dA 1.10 × 10^1^ ± 6.83)^28^,^29^,^30^,^31^,^32^.

## Discussion

The assimilation of ^15^N_2_ gas into tissues of *Agave* seedlings is indicative of the presence of nitrogen-fixing microorganisms within seedling tissues. From seedlings, seeds and bulbillos, we consistently isolated *Bacillus tequilensis*; however, plants may also have contained other non-cultured bacteria that could have been responsible for N fixation in plant tissues. We do not consider *B. tequilensis* to be responsible for N_2_ fixation in plant tissues; however, because of its ease of culturing and its endophytic nature, we employed *B. tequilensis* in experiments to evaluate the hypothesis that nitrogenous nutrients may flow from bacterial endophyte populations to plants.

We labeled proteins and nucleic acids of *B. tequilensis* with ^15^N; then inoculated plants with suspensions of bacteria. Using these bacteria we conducted two experiments. In the first experiment we watered plants weekly with a 4 mL suspension of 80•10^6^ CFU mL^−1^ of ^15^N-labeled *Bacillus* for a six-month period. Our results show that plants incubated with the bacterial endophyte came to contain N that was originally contained within the bacterium. Detection of ^15^N-labeled tryptophan and 2′-deoxynucleosides suggests that ^15^N-labeled bacterial endophytes enter into or otherwise associate with plant tissues. Because both bacteria and plants possess tryptophan and 2′-deoxynucleosides, with this data alone we cannot evaluate transfer of nitrogen from bacterium to plant. However, we were able to detect the ^15^N in pheophytin *a*, a molecule derived from plant chlorophylls. Because chlorophyll is unique to the plant cells, the presence of the ^15^N label there is a definitive confirmation of transference of N from bacterium to plant tissues.

A second experiment was conducted to evaluate whether soil absorption from dead bacteria could account for some ^15^N movement into plant tissues, we developed an experiment in which we treated plants with live or heat-killed ^15^N-labeled bacteria (121°C, 10 min); and with MMN solution, non-endophytic *E. coli* (not labeled) and water as controls. Plants were watered weekly for a two-month period using 4 mL of bacterial suspension at a concentration of 80•10^6^ CFU mL^−1^or MMN solution. In this experiment we found significantly more incorporation of ^15^N into 2′-deoxynucleosides in plants treated with live *Bacillus* than in plants treated with heat-killed *Bacillus*. We further evaluated the growth of these plants compared with those treated with water, *E. coli* or mineral nutrient solution. The amount of total acquired biomass was almost double in plants treated with living *Bacillus* than in plants treated with heat-killed *Bacillus* and *E. coli*; and more than 3.3 times that seen in plants treated with mineral solution. Enhanced growth of plants, and incorporation of the ^15^N label into plants treated with living ^15^N-labeled bacteria are results that are consistent with a scenario where N is transferred to plants from bacteria within tissues of plants rather than being absorbed primarily from dead bacteria in the soil. The fact that non-endophytic *E. coli*-treated plants demonstrated a biomass increase half that of living endophyte cultures suggests that efficient movement of N from bacteria to plants is a function of living, plant-colonizing, endophytic bacteria. The results of our experiments suggest that some nitrogen may come from decomposition of microbes in the soil. Decomposition of microbes in the soil could explain biomass increases in plants treated with heat-killed *B. tequilensis* and non-endophytic *E. coli*.

Microscopic observations of plant roots treated with the living *Bacillus* and stained using a reactive oxygen probe showed that degrading bacterial cells were associated with reactive oxygen and were often internalized into plant roots. Our observations are similar to those of Paungfoo-Lonhienne et al.^3^, where microbes were shown to enter into root cells of tomato and *Arabidopsis* where they were degraded. The involvement of reactive oxygen in procurement of organic nutrients from bacteria is consistent with ‘oxidative nitrogen scavenging', where reactive oxygen and proteases may be involved in nutrient extraction from bacteria^4^,^33^. We did not visualize bacteria or their degradation in shoot tissues; however, this is likely due to failure of the aqueous stains to penetrate into the shoot, rather than their absence from shoots.

In a recent study that evaluated the contribution of organic N in wheat from direct microorganism consumption against nitrate, *L*-alanine and *L*-tetraalanine absorption, it was found that plants absorbed nutrients through endocytosis of soil microbes, but the N obtained through this process was up to two orders of magnitude slower than other forms of organic and inorganic N in the soil^5^. Our study examining nutrient transfer from *B. tequilensis* to *Agave* suggests a process where endophytic bacterial degradation may supply N for plant growth; however, this mechanism may not be rapid and it could depend on occurrence of N deficiency or other nutrient depletion in soils. Our initial experiment to evaluate movement of ^15^N into plant molecules lasted six months. We do not know the minimum amount of time or precise plant growth conditions needed to see movement of N from bacterial endophytes to host plants. We propose that efficiency of this mode of nutrition depends on the particular host and microbe association. *Agave tequilana* is a desert plant adapted to growth under low N soil conditions and *B. tequilensis* is a native endophytic microbe of that plant. Internal colonization of plant tissues may increase the probability of transference of nutrients from endophytes to host plants. In this respect endophytic microbes that fix N may represent a nutritional resource that may be tapped into when soil N is limiting. Equally important to plants could be N derived from soil microbes that grow and obtain nutrients in the soil then colonize growing plants where they may be degraded. Whether N derived from microbes is a significant source of N for plants is a question that requires additional investigation. While our experiments on N transfer from endophyte to host in *Agave* are not exhaustive, they do provide further evidence that plants may obtain N through degradation of symbiotic microbes^3^,^4^,^5^,^33^.

## Methods

### Plant materials

For experiments we used one-yr-old seeds and asexual plantlets (bulbillos) derived from plants of *Agave tequilana* Weber that were originally collected on an *A. tequilana* plantation near Atotonilco el Alto, Jalisco, Mexico at coordinates 20°34′22.71″N, 102°32′00.85, 1900masl.

### ^15^N_2_ gas assimilation experiment with seedlings

An experiment was conducted to evaluate whether endophytic microbes fix nitrogen within intact plant tissues. In this experiment seeds were surface disinfected in 3% sodium hypochlorite for 20 min with constant agitation to remove external bacteria, then rinsed three times using sterile water. Five seeds were plated onto 0.7% agarose media in each of eight Petri dishes. Four Petri plates were placed in a 1-liter gas chamber in which the air was enriched with 33 mL of ^15^N_2_ gas. The other four Petri dishes were placed in a chamber in which the air was not enriched with ^15^N_2_. Both chambers were placed under fluorescent lighting with alternating light/dark periods (10 hr/14 hr) at laboratory ambient temperature for 21 days. After incubation shoots were excised from roots, washed to remove any superficial bacteria, then dried for 14 hr in an oven at 60°C. All shoots from a plate were combined to ensure sufficient material for analysis. For mass-spectroscopic ^14^N/^15^N ratio analysis, we sent 0.9–1.0 g of dried shoot material to the Stable Isotope/Soil Biology Laboratory at the Odum School of Ecology at the University of Georgia, Athens, USA.

### Isolation of bacteria from *A. tequilana* seeds

Seeds were surface sterilized to remove epiphytic microbes. In this process seeds were immersed in a 3% hypochlorite solution for twenty minutes with constant agitation. The seeds were then rinsed with sterile distilled water and placed in 85% ethyl alcohol for ten minutes, followed by three rinses with sterile distilled water. To confirm the disinfection process, aliquots of the sterile water used in the final rinse were plated in tryptic soy agar (TSA; Difco, Sparks, MD, USA) and incubated at 28°C for 15 days, after which plates were examined for the presence of microorganism growth. To isolate bacteria, we used two procedures: 1) 30 sterilized seeds were placed onto 15 cm diameter Petri plates containing potato dextrose agar (PDA, Difco, Sparks, MD); and 2) Ten seeds were ground with 5 mL of aqueous solution (0.9% NaCl) using a sterile mortar and pestle and the seed extract was plated on TSA plates with different volumes (50 to 350 microliters per plate). All plates were incubated at 28°C for seven days until cream-colored colonies appeared.

### Identification of bacterium by 16S rRNA and sequencing

For bacterial identification, 980 base pairs of the 16S rDNA region were sequenced^24^. Sequences were compared to sequences available in the NCBI GenBank database to identify the closest matches. We submitted a representative sequence to NCBI (GenBank accession number KF792125).

## Experiment 1

### Plant growth conditions

To reactivate bulbillos of *A. tequilana* collected from the field, plants were grown in sterile soil in a growth chamber at 27°C with 50% humidity under 14 h light and 10 h dark photoperiods and lamp intensities of 80 Watts/m^2^ for three weeks. Then plantlets were rinsed with sterile water to eliminate organic soil on roots and transferred immediately to N-free sand (30 g per glass bottle) for another three weeks maintained under the same conditions.

### Visualization of bacterial oxidation in *A. tequilana* seedlings and bulbillos

To visualize bacterial oxidation in plant tissues, we stained seedlings and bulbillos with DAB/horseradish peroxidase for a 6–12 h period. We then excised seedling roots and shoots and placed them on a slide containing 0.1% aqueous toluidine blue. The DAB/peroxidase probe was prepared with a 5 mL solution of 100 mM potassium phosphate buffer, pH 6.9, 2.5 mM diaminobenzidine tetrachloride and 5-purpurogallin units/mL of horseradish peroxidase (Type VI, Sigma, St. Louis, MO, USA)^25^,^33^. Slides were examined using bright field microscopy on a Zeiss Axioskope® with a Spot Insight™ 4 megapixel digital camera.

### Bacterial growth and [^15^N]-labeling

*Bacillus tequilensis* was grown and maintained in TSA. To evaluate transferral of organic N from bacteria to plants under limited nutrient conditions, we labeled *B. tequilensis* using ^14^NH_4_Cl or ^15^NH_4_Cl as the sole N sources in minimal medium M9 and incubated for 18 h at 200 rpm. All bacteria cultured with ^15^N contained nucleic acids labeled with isotopic N (Supplementary Fig. S8). ^15^N-Labeling of *Bacillus tequilensis* was confirmed by reversed phase HPLC-MS analysis of DNA bases (Supplementary Fig. S8). The HPLC-MS results showed complete ^15^N-labeling of nitrogen atoms of purine and pyrimidine bases through detection of ^15^N-labeled guanine at *m/z* 157 (Supplementary Fig. S8B), ^15^N-labeled cytosine at *m/z* 115 (Supplementary Fig. S8C), ^15^N-labeled adenine at *m/z* 157 (Supplementary Fig. S8D) and ^15^N-labeled thymine at *m/z* 129 (Supplementary Fig. S8E). Agave plantlets growing in sterile sand (SiO_2_) were inoculated with ^15^N-labeled *B. tequilensis* (^15^N-Bteq) or unlabeled *B. tequilensis* (^14^N-Bteq). Four mL of bacteria adjusted to 1 at OD_600_ (equivalent to 80•10^6^ CFU mL^−1^) were administered to plants every week for six months. Controls included plants treated with H_2_O, ^14^N and ^15^N MMN solution.

### Plant treatments and inoculation with bacteria

The plants used in this study were treated with 4 mL of: 1) water, 2) unlabeled MMN medium containing NH_4_Cl (^14^NH_4_Cl), 3) labeled MMN medium containing ^15^NH_4_Cl (^15^NH_4_Cl), 4) unlabeled bacteria suspension (^14^N*-Bteq*) or 5.) labeled bacteria suspension (^15^N*-Bteq*). NH_4_Cl medium or ^15^NH_4_Cl medium was prepared with 0.5 g/L ^14^NH_4_Cl or ^15^NH_4_Cl and 0.05 g/L CaCl_2_, 0.025 g/L NaCl, 0.05 g/L KH_2_PO_4_, 0.15 g/L MgSO_4_.7H_2_O, 1 mg/L FeCl_3_.6H_2_O, 5 g/L glucose monohydrate and 10 mL of trace elements solution. Trace element solution contained 3.728 g/L KCl, 1.546 g/L H_3_BO_3_, 0.845 g/L MnSO_4_.H_2_O, 0.05 g/L ZnSO_4_.7H_2_O, 0.0125 g/L CuSO_4_ and 0.05 g/L (NH_4_)_6_Mo_7_O_24_.4H_2_O. *Bacillus tequilensis* cells were grown for 18 h at 37°C in minimal medium with ^14^N and ^15^N as NH_4_Cl, pelleted by centrifugation (5,000 × gravity, 20 min, 4°C), and washed three times in sterilized glucose solution (0.08%). Four-mL of labeled or unlabeled bacterial cells (see above section ‘Bacterial growth and ^15^N labeling') were adjusted to 1 at OD_600_ (equivalent to 80•10^6^ CFU mL^−1^) and used to inoculate each plant of *A. tequilana* maintained in sterile sand (30 g/dry wt). Additional treatments included plants watered with a mineral solution (50% MMN) supplemented with isotopic or non-isotopic NH_4_Cl; and plants watered with sterilized distilled water. The plants were watered once a week with 4 mL of each treatment during a six-month period. All plants were approximately 10–12 cm in height with 1 or 2 open leaves. Plants were grown in glass bottles in a growth chamber at 27°C day/night temperature, 14 h photoperiod.

### Analysis of plant for ^15^N incorporation

The central leaf ‘cogollo' and new leaves of *B. tequilensis*-inoculated plants were taken and the presence of ^15^N-labeled tryptophan (^15^N-Trp) was quantified by High-Performance Liquid Chromatography coupled to Mass Spectrometry in tandem (HPLC-MS/MS). To reduce the chances that the ^15^N-labeled tryptophan was from the living bacterium rather than the host plant*,* the extraction was carried out seven days after inoculation with ^15^N-labeled bacteria. The detection and quantification of ^15^N-Trp in *A. tequilana* was performed by HPLC-MS/MS, in the Selected Reaction Monitoring (SRM) mode (*m/z* 207→189) (Supplementary Fig. S2–S4). The concentration of ^15^ N-Trp was determined in the samples through a calibration curve with standard solution of ^15^ N-Trp and melatonin D_3_-labeled (Mel-D_3_), as internal standard (Supplementary Fig. S5–S7)^28^.

### Protein extraction from *A. tequilana*

See Supplementary Note 1.

### HPLC-MS/MS analysis of tryptophan

See Supplementary Note 2.

### Extraction of pheophytin from *A. tequilana*

To confirm that N transferred from microbes to plant tissue, an analysis in high-resolution mass spectrometry was carried out to evaluate the relative abundance of isotopomers of pheophytin (Fig. 4). Pheophytin is a magnesium-free derivative of chlorophyll, which has advantages over chlorophylls for isotopic analysis because of its stability, simpler mass spectra and better ionization characteristics. Pheophytin was extracted following the method of Perkins and Roberts^26^,^27^, with some modifications. Briefly, 0.5 g fresh leaves were frozen in liquid N_2_ and ground to a fine powder using mortar and pestle. For pheophytin extraction equal volumes of 85% acetone in 1 mL water, and ethyl ether were added and centrifuged at 10, 000 rpm for 10 min. The ether phase was collected and the procedure was repeated two times. Chlorophyll was converted to pheophytin by adding 10 µL diluted 6 M HCl to the ether extract. The excess of HCl was removed by adding 500 µL of water and centrifuging at 10, 000 rpm for 10 min, three times. The ether phase was collected, dried and prepared for HPLC/MS analysis by adding methanol to obtain a final sample concentration of 1 mg/mL.

### HPLC/MS analysis of pheophytin

Pheophytin samples were analyzed using a MicroTOFQ-II mass spectrometer (Bruker) coupled to a Shimadzu HPLC system (Tokyo, Japan) with two pumps LC-20AD, automatic injector SIL-20A, column oven CTO-20A, UV detector SPD-20A and controller CBM-10A. A column Phenomenex Luna 5 µm (PFP2 150 × 2 mm, 100A particle size) was used and chromatography was performed with a flux of 200 µL/min using acetonitrile:H_2_O (+ 0.1% formic acid) as mobile phase in a gradient of 0 to 5 min 60% of acetonitrile, from 5 to 30 min 60 to 100% of acetonitrile. The column oven was kept in 40°C, UV detector was recorded at 400 and 600 nm. The mass spectrophotometer was operating in electrospray positive mode, with a nebulization and drying gas at 4 Bar and 8 L/min, respectively. Capillar voltage was set to 4500 V and drying temperature in 200°C. Collision cell and quadrupole energy were set to 20 eV and 10 eV, respectively. The molecular formula of pheophytin *a* is C_55_H_74_N_4_O_5_ and the base peak was detected at [M+H]^+^ = 872.5731.

### Extraction of DNA from *A. tequilana*

DNA extraction from *A. tequilana* leaves was made after six months of treatments as described above. Freshly collected leaves (1 g) were ground to powder in liquid nitrogen and DNA was extracted with 1 mL of 50 mM extraction buffer (pH 8.0) containing 2% CTAB (p/v), 1.4 M NaCl, 100 mM Tris-HCl, 20 mM EDTA and 0.2% (v/v) β-mercaptoethanol. The solution was kept at 60°C, 800 × r.p.m for 30 minutes. Following, eppendorf tubes were left at room temperature and 500 µl of chloroform: isoamyl alcohol (24:1 v/v, −20°C) was added. The solution was maintained under agitation (10 minutes, 300 × r.p.m), centrifuged (12, 000 × r.p.m, 10 min, room temperature) and the organic phase discarded. RNAse A was added (in Tris 50 mM, pH 8.0, final concentration: 0.1 mg/mL) and the solution was incubated at 37°C for 30 minutes and agitated at 300 × r.p.m. Following this, isopropyl alcohol (−20°C) was added and the mixture was gently shaken and centrifuged (20 minute, 13,000 × r.p.m). This procedure was repeated twice. The mixture was washed with 60% isopropyl alcohol (−20°C) twice, centrifuged (5 min, 3,000 × r.p.m) and supernatant discarded. The DNA pellet was washed twice with 500 µL of 70% ethanol (−20°C), centrifuged, dried on speed vac and re-suspended in 0.1 mM desferroxamine. DNA concentration was measured spectrophotometrically at 260 nm.

### Enzymatic hydrolysis of DNA from *A. tequilana*

See Supplementary Note 3.

### HPLC-MS/MS analysis of DNA from *A. tequilana*

HPLC-MS/MS analysis was performed using an Agilent HPLC (1200 series, Agilent Waldbronn, Germany) coupled to a linear ion trap mass spectrometer (4000 QTRAP mass spectrometer, Applied Biosystems, Foster City, CA, USA) with electrospray ionization source. The column oven and auto sampler temperatures were set at 25°C and 4°C, respectively. For the separation, a reversed phase column was used [(C18(2)-HST Luna, Phenomenex, 100 mm × 2.0 mm, 2.5 μm particle size)]. Flow rate was set at 0.2 mL/min. Gradient elution was carried out with 0.1% formic acid (A) and acetonitrile:0.1% formic acid (8:2, v/v) (B). The separation was conducted with 0 to 40% B during the first 15 minutes, 40% B for 10 minutes, 40% to 0% B for 1 minute and 0% B for 35 minutes. MS/MS spectrometry analysis was performed in positive ionization mode and using SRM mode for each 2′-deoxynucleoside that corresponded to the loss of 2-deoxyribose moiety: 2′-deoxyguanosine (^15^N_5_-dG, *m/z* 273→157), 2′-deoxycytidine (^15^N_3_-dC, *m/z* 231→115), 2′-deoxyadenosine (^15^N_5_-dA, *m/z* 257→141), 2′-deoxythymidine (^15^N_2_-dT, *m/z* 245→129), 5-methyl-2′-deoxycytidine (^15^N_3_- MedC, *m/z* 245→129) and *N*′-methyl-2′-deoxyadenosine (^15^N_5_-MedA, *m/z* 271→155) (Fig. 3 and Supplementary Fig. S9 to 11)^29^,^30^,^31^,^32^. Mass spectrometry analyses were performed with the following parameters: collision excitation potential, 10 V; collision activated dissociation gas flow, medium; pause time, 5 ms; dwell time, 300 ms; curtain gas, 12 psi; ion source, 5500 V; temperature, 650°C; gas 1 and gas 2, 40 psi; declustering potential, 31 V and entrance potential, 10 V.

Analysis of ^15^N_3_-dC and ^15^N_5_-dA extracted from DNA of plants treated for two months with live or heat-killed ^15^N-labeled bacteria was performed as described above.

### HPLC-MS analysis of DNA from bacteria

See Supplementary Note 4.

## Experiment 2

### Plant mass accumulation study

To evaluate whether soil absorption from dead bacteria could account for some ^15^N movement into plant tissues, we developed an experiment in which we treated plants (six replicates per treatment) over a two month period with live or heat-killed ^15^N-labeled *B. tequilensis* (heated to 121°C, 10 min), and with 50% MMN solution, non-endophytic *E. coli* (not labeled) and water as controls. Bacteria were applied as described in Experiment 1. To assess increase of ^15^N in leaves of Agave plants treated with ^15^N-labeled bacteria, deoxynucleosides were extracted from leaves and 2′-deoxycytidine and 2′-deoxyadenosine were measured. Measurements of plant mass with water-washed roots were made before and after two months of treatments. Biomass increase is the change in whole plant wet weight as a result of the treatments.

### Statistical analyses

Statistical tests were performed with Origin (version 8.0) and GraphPad PRISM (version 5.0) programs. Significant differences were determined by t-test and one-way ANOVA (applying Dunnett's post-test) with the level of significance set at P < 0.05 for ^15^N-Trp quantification, detection of nucleosides (^15^N_3_-dC and ^15^N_5_-dA) and biomass increase, respectively.

## Author Contributions

M.J.B.-G., P.D.M. & J.F.W. developed the concept of the experiments and analyses. M.J.B.-G., F.M.P., K.R.P. and M.S.T. implemented experiments. F.M.P., K.R.P., M.H.G.M., L.F.Y. & M.J.K. conducted biochemical analyses. M.S.T. isolated and identified bacteria. All authors contributed to data interpretation and writing of the manuscript.

## Supplementary Material

Supplementary Information SUPPLEMENTARY INFORMATION Nitrogen acquisition in Agave tequilana from degradation of endophytic bacteria
