# Supplementary material for: Nitrogen acquisition in Agave tequilana from degradation of endophytic bacteria
Source: Sci Rep. 2014 Nov 6;4:6938. doi: 10.1038/srep06938 (PMC4221784; doi:10.1038/srep06938)
Supplement: Supplementary Information — Nitrogen acquisition in Agave tequilana from degradation of endophytic bacteria [file srep06938-s1.doc]

SUPPLEMENTARY INFORMATION

**Nitrogen acquisition in *Agave tequilana* from degradation of endophytic bacteria**

Miguel J. Beltran-Garcia^1, 3^, James F. White Jr^2^, Fernanda M. Prado^3^, Katia R. Prieto^3^, Lydia F. Yamaguchi^4^, Monica S. Torres^2^, Massuo J. Kato^4^, Marisa H. G. Medeiros^3^ & Paolo Di Mascio^3^

**^1^** Departamento de Química ICET, Universidad Autonoma de Guadalajara, Patria 1201, Lomas del Valle, Zapopan Jalisco, Mexico.

^2^ Department of Plant Biology and Pathology, Rutgers University, New Brunswick, NJ USA.

^3^Departamento de Bioquímica, Instituto de Química, Universidade de São Paulo, 05508-000, São Paulo, SP, Brazil.

^4^Departamento de Química Fundamental, Instituto de Química, Universidade de São Paulo, 05508-000, São Paulo, SP, Brazil.

James F. White ([white@rci.rutgers.edu](mailto:white@rci.rutgers.edu)) & Paolo Di Mascio ([pdmascio@iq.usp.br](mailto:pdmascio@iq.usp.br))

**Supplementary Note 1.**

**Protein extraction from *A. tequilana.***

Protein extraction from the leaves of *A. tequilana* was made after six months of treatments as described above. Freshly collected leaves (90-140.0 mg) were ground to powder in liquid nitrogen, washed with 1mL of 50 mM phosphate buffer (pH 6.0) and centrifuged at 12, 000 x gravity for 20 min at room temperature. Proteins were precipitated adding 2.4M (NH_4_)_2_SO_4_ (600µL), followed by agitation (10 °C during 30 min), and then the solution was centrifuged (10, 000 x gravity, 4 ºC for 10 min). In the final step 600 µL of 6 M HCl was added, and solution was maintained under agitation for 24 h at 90 ºC. The solvent was dried in a Speed Vac, after that 500µL of water was added prior HPLC-MS/MS analysis.

**Supplementary Note 2.**

**HPLC-MS/MS analysis of tryptophan.**

HPLC-MS/MS analysis was performed in an Agilent HPLC (1200 series, Agilent Waldbronn, Germany). The column oven and auto sampler temperatures were set at 25 °C and 4 °C, respectively. For the separation, a C18 column was used (250 mm x 4.6 mm, 5μm particle size, Gemini Phenomenex, Torrance, California, USA) and flow rate was set at 1.0 mL/min. Gradient elution was carried out with 0.1 % formic acid (A) and acetonitrile (B). The separation was conducted with 0 % B during first 5 min, 0 to 60 % B for 15 min, 60 % B for 5 min, 60 to 0 % B for 25.5 min and 0 % B for 30 min. Samples of 100 µL were injected. For the MS/MS analysis, it was used a 4000 QTRAP mass spectrometer (Applied Biosystems, Foster City, CA, USA) with an electrospray ionization source. The samples were analyzed in the positive ion mode by an Enhanced Mass Spectrum (EMS), Enhanced Product Ion (EPI), and Selected Reaction Monitoring (SRM) mode. Isotopically [^15^N]-labeled tryptophan (^15^N-Trp) was detected and quantified by SRM mode, using melatonin isotopically D_3_-labeled (Mel-D_3_) as internal standard ^24^. The SRM detection of the ^15^N-Trp and Mel-D_3_ was performed by the mass transitions *m*/*z* 207
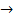
189 and 236
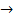
174, respectively. A standard calibration curve for ^15^N-Trp was prepared using six aliquots of increasing concentrations (0, 0.03, 0.75, 1.5, 3.0 and 6.0 nM) and Mel-D_3_ as internal standard in the final concentration of 50 nM. The SRM analyses were performed using collision energy of 17eV for ^15^N-Trp and 23eV for Mel-D_3_. Collision excitation potential was 10V and 8V for ^15^N-Trp and Mel-D_3_, respectively. The collision activated dissociation (CAD) gas flow was set as medium. Each transition was obtained with 5 ms of pause time and a dwell time of 200 ms. The information dependent acquisition (IDA) scan intensity threshold was set at 500, 000 counts per seconds (cps). EMS survey scan was acquired with a scan rate of 1000 amu/s. The CAD gas flow was adjusted as medium and the mass range was set at 190-250 *m*/*z*. EPI scan analyses of ^15^N-Trp (*m/z* 207) and Mel-D_3_ (*m/z* 236) were performed with CE and collision excitation spread at 15eV and 10 eV, respectively. The dynamic fill time was used, setting first the linear ion trap (LIT) fill time at 20ms. The CAD gas flow for EPI experiments was adjusted as low and the mass range was set at 100-240 *m*/*z*. The source parameters were set as follows: curtain gas at 10 psi; ion source at 5500 V; temperature at 700°C; gas 1 and gas 2 at 50 psi; declustering potential at 51V and entrance potential at 10V.

**Supplementary Note 3.**

**Enzymatic hydrolysis of DNA from *A. tequilana.***

Two microliters of 3 M sodium acetate buffer (pH 5.0) were added to a solution (50 µL) containing approximately 15.0 µg/mL of DNA extracted and 0.1 mM of desferroxamine. DNA was digested with 2.4 units of nuclease P1 (0.4 U/µL) at 37 °C, 300 rpm for 30 min. Then, 2 µL of 3 M Tris-HCl buffer (pH 7.5) and 12 units of alkaline phosphatase (2 U/µL) were added for additional incubation (1 hour, 37 ºC, 300 x r.p.m). The final volume of solution was adjusted to 50 µL with methanol and 20 µL of each sample was injected into HPLC-MS/MS system.

**Supplementary Note 4.**

**HPLC-MS analysis DNA from bacteria.**

Bacterial DNA was extracted following a short protocol proposed by Wilson, K *(22)*. DNA was hydrolyzed with 6 M HCl (2 h at 65 °C), neutralized with 25 % ammonia solution and analysed by HPLC-MS. HPLC-MS analyses were carried out in a Shimadzu HPLC system (Tokyo, Japan) coupled to a Quattro II mass spectrometer (Micromass, Manchester, UK) with a Z-spray source. Mass spectrometry analyses were performed in the positive mode with source temperature at 150°C, desolvation temperature at 200 °C, capillary voltage at 4.0 kV, sample cone voltage and extractor cone voltage at 30 V and 5 V, respectively. For analytical purposes a Phenomenex Gemini C-18 column (250 × 4.6mm i.d., 5 μm particle size) was used with the UV detector set at 260 nm. DNA bases were separated using the mobile phase 2 mM ammonium formate (A), acetonitrile (B) and flow rate of 0.6 mL/min. The linear gradient was 0 %B during 5 min, 0 to 20 %B over 15 min, 20 to 100 % B over 5 min, 100 %B for 5 min, returning to 0 % B over 2 min and maintaining 0 %B until 35 min. The column oven was set at 25 °C. Flux directed to the mass spectrometer was 0.135 mL/min and full-scan data were acquired over a mass range of 100–600 *m/z*.

**Fig. S1 |** **Quantification of ^15^N-labeled tryptophan (^15^N-Trp) in foliar tissue of *A. tequilana* using HPLC-MS/MS by SRM mode.** The sample groups analyzed were H_2_O treated, ^14^NH_4_Cl and ^15^NH_4_Cl. The quantity of ^15^N-Trp (ng/mg) represents the average of two different samples.

Fig. S2 | HPLC-MS/MS detection of ^14^N-Trp and ^15^N-Trp. Injection of 10µL of 5µM ^14^N-Trp/^15^N-Trp standard solution, (A) EMS of ^14^N-Trp and ^15^N-Trp, (B) EPI mass spectrum of ^14^N-Trp (*m/z* 205) and (C) EPI mass spectrum of ^15^ N-Trp (*m/z* 207).

Fig. S3 | HPLC-MS/MS detection of Mel-D_3_. (A) EMS and (B) EPI mass spectrum of 5µM Mel-D_3_ (*m/z* 236), injection of 10µL.

Fig. S4 | HPLC-MS/MS detection of ^14^N-Trp and ^15^N-Trp. ^14^N-Trp (*m/z* 205→188) (A), ^15^N-Trp (*m/z* 207→189) (B) and Mel-D_3_ (*m/z* 236→174) (C) in a mixture standard solution (5µM final concentration and 10µL injected).

**Fig. S5** | **HPLC-MS/MS detection of ^15^N-Trp.** SRM mode (*m/z* 207→189) in the samples ^15^N*-Bteq* (A), ^14^N*-Bteq* (B) and H_2_O (C). Data represents the mean values ± standard error of the mean from three independent experiments. (*)^15^N-labeled *B. tequilensis* data are significantly different when compared to the H_2_O and unlabeled *B. tequilensis* groups (p<0.05; t-test).

Fig. S6 | HPLC-MS/MS detection of ^15^N-Trp. SRM mode (*m/z* 207→189) in the samples ^15^NH_4_Cl (A), ^14^NH_4_Cl (B) and H_2_O (C).

Fig. S7 | HPLC-MS/MS detection of ^15^N-Trp. SRM mode using the mass transition *m/z* 207→189 for ^15^N-Trp (A) and *m/z* 236→174 for the internal standard Mel-D_3_ (B). Insert: Calibration curve of ^15^N-Trp with increased concentrations of ^15^N-Trp (0, 0.03, 0.75, 1.5, 3.0 and 6.0 nM) vs 50 nM Mel-D_3_. Mel-D_3_ was synthesized according to Almeida et al. (28). Mel-D_3_ was purified by a HPLC system consisting of two LC-10ADVP pumps, a SPD-M10AVP photodiode array with wavelength setting at 280nm, SCL-10AVP system controller, and monitored by Class-VP 5.032 software (Shimadzu, Kyoto, Japan). A semi-preparative Luna 10 C-18 (2) column (250 x 10 mm, 5µm particle size) was used with flow rate of 4 mL/min. Water (A) and methanol (B) were used in mobile phase with linear gradient of 30–70% B for 10 min, 70% B for 10 min, 70-30% B during 1 min and 30% B for 25 min.

Fig. S8 | HPLC-MS analysis of DNA. *B. tequilensis* cultivated in a medium with ^15^NH_4_Cl. UV chromatogram at 260nm (A). Mass spectrum obtained from peak at 15.82 (^15^N-labeled guanine) (B), 8.24 (^15^N-labeled cytosine) (C), 17.81 (^15^N-labeled adenine) (D) and 16.73 minutes (^15^N-labeled thymine) (E).

**Fig. S9 | HPLC-MS/MS analysis of ^15^N-labeled 2´-deoxynucleosides from *A. tequilana* supplemented with ^15^NH_4_Cl.**  Using the SRM mode, 2´-deoxynucleosides were detected by the loss of 2-deoxyribose moiety: ^15^N_5_-dG, *m/z* 273→157 (A), ^15^N_3_-dC, *m/z* 231→115 (B), ^15^N_5_-dA, *m/z* 257→141 (C) and ^15^N_2_-dT, *m/z* 245→129 (D).

**Fig. S10 | HPLC-MS/MS analysis of ^15^N-labeled 2´-deoxynucleosides from *A. tequilana* supplemented with ^15^N-labeled *B. tequilensis.*** Using SRM mode, 2´-deoxynucleosides were detected by the loss of 2-deoxyribose moiety: ^15^N_3_- MedC, *m/z* 245→129 (A) and ^15^N_5_-MedAdo, *m/z* 271→155 (B). Peaks at a and b correspond to ^15^N_2_-dT and ^15^N_5_-dG, respectively.

**Fig. S11 | HPLC-MS/MS analysis of ^15^N-labeled 2´-deoxynucleosides from *A. tequilana* supplemented with ^15^NH_4_Cl**. Using SRM mode, 2´-deoxynucleosides were detected by the loss of 2-deoxyribose moiety: ^15^N_3_- MedC, *m/z* 245→129 (A) and ^15^N_5_-MedAdo, *m/z* 271→155 (B). Peaks at a and b correspond to ^15^N_2_-dT and ^15^N_5_-dG, respectively.
